# The Influence of Therapist Adherence on Multisystemic Therapy Treatment Outcome for Adolescents with Antisocial Behaviours: A Retrospective Study in Western Australian Families

**DOI:** 10.3390/ijerph22081310

**Published:** 2025-08-21

**Authors:** Leartluk Nuntavisit, Mark Robert Porter

**Affiliations:** Multisystemic Therapy Program Specialised Child and Adolescent Mental Health Service (CAMHS), Department of Health, Fremantle Hospital, Fremantle, WA 6160, Australia; mark.porter@health.wa.gov.au

**Keywords:** multisystemic therapy, conduct disorder, antisocial behaviour, therapist adherence, treatment fidelity

## Abstract

Multisystemic Therapy (MST) is an intensive family and community-based treatment targeting antisocial behaviours in adolescents. Treatment fidelity has proved crucial for successful implementation of the MST intervention, with prior research demonstrating a strong association with positive and enduring treatment outcomes. The Therapist Adherence Measure (TAM) is a standardised measure reported by caregivers and comprised of 28 items based on the nine treatment principles of MST. Several randomised control trials have confirmed that therapist adherence to the MST model is a valid predictor for a reduction of antisocial behaviours in adolescents. However, there is limited understanding of mechanisms by which therapist model adherence is related to positive changes in family relations and association with decreased adolescent behavioural problems. In this retrospective study, we evaluated effects of therapist adherence on changes in parental factors (e.g., parental mental well-being, monitoring and discipline approach) which in turn were associated with decreased behavioural problems in adolescents. We extracted data collected from 186 families engaged with the MST research program operating within the Western Australian Child and Adolescent Mental Health Service (CAMHS) during 2018–2024. Data for TAMs were collected monthly during treatment, and family outcome measures were collected at pre-treatment and post-treatment. The finding highlights the importance of therapists maintaining treatment fidelity and addressing treatment barriers throughout MST intervention to ensure the desired therapeutic outcomes.

## 1. Introduction

Conduct disorder (CD) is one of most common mental and behavioural problems in children and adolescents [1] and ranked in the top five of leading causes of the total burden of disease in Australian children [2]. Conduct disorders are a psychiatric disorder diagnosed in children and adolescents and characterized by a pattern of behaviours ranging from non-compliance, hostile and aggressive behaviours, to severe antisocial and law-breaking behaviours. A 2015 report from the Mental Health of Australian Children and Adolescents Survey [3] indicated that children with conduct disorder ranked second in service utilisation among all children with mental disorders. Additionally, the report noted that two thirds of children and adolescents with conduct disorder experience concurrent mental disorders, e.g., ADHD and mood disorders, which require a more comprehensive treatment approach. Children with oppositional behaviours are argumentative, hostile and defiant, and if untreated, they are more likely to develop a conduct disorder exhibiting a range of delinquent behaviours including physical aggression, absconding from home and school, deliberately destroying other’s property, theft and/or robbery, fire-setting, animal cruelty and substance use. Conduct disorder negatively affects the well-being of both children and family members who are likely to experience the repercussions of the child’s behaviours, e.g., verbal and physical assault in their own home, police involvement or legal sanctions and social exclusion. In the absence of effective intervention, conduct disorder is a strong predictor of various adult mental illness, substance abuse, chronic unemployment, domestic violence and incarceration [4]. Many of these difficulties are chronic and very costly to families and the wider community. To alter the likelihood of these detrimental life trajectories, factors that influence children’s emotional and behavioural problems need to be identified and integrated into interventions.

Research has shown many young individuals diagnosed with conduct disorder often have a history of or current adverse childhood experiences. These can include various forms of trauma such as abuse, neglect, dysfunctional household, family and community violence. Children with a high risk for antisocial behaviours and mental health disorders have often experienced childhood traumas, poverty, family disruptions and abuse [5,6], and are more likely to have caregivers with various difficulties including unemployment, substance misuse, domestic violence and chronic engagement with adult mental health services [7]. These difficulties typically impact on the caregiver’s ability to parent effectively [8] and several studies have shown parent mental well-being as a key contributor to effective parenting [9,10,11]. Lack of effective parenting has been associated with adolescent mental health problems, substance misuse, school disengagement and juvenile offending [12,13]. Effective parenting requires caregiver’s warmth and absence of hostility, the establishment of clear rules and expectations, and consistent involvement in the child’s life [14,15]. Therefore, interventions which aim to strengthen parent–child connection should identify the protective factors such as the caregiver’s health and mental well-being, as this will affect the caregiver’s ability to provide appropriate warmth and care for the children.

The MST treatment model was designed by Scott Henggeler in the 1970’s and after years of subsequent robust research, the model developed practice guidelines. The intervention was designed to target adolescents presenting with serious problem behaviours, particularly violent and antisocial behaviours. MST is an intensive family and community-based intervention based on the social ecological theory of human behaviour proposed by Bronfenbrenner (1979). The theory examines the complex systems that interplay between personal, interpersonal, institutional, societal and community factors which influence human behaviours [16]. MST analyses the inter-connected systems surrounding a young person, and identifies the most influential factors that will reduce a young person’s emotional and behavioural problems when modified [17]. MST interventions utilise a range of empirically validated treatments including cognitive behaviour therapy, parental skills training and structural family therapy, whilst incorporating frameworks from family systems theory [18] and social ecological theory [16]. The role of parent–child interaction has a strong influence on the psychological, emotional and social well-being of children and adolescents that has been highlighted in several theoretical works. Enhancing interpersonal relationship and social supports within an adolescent’s social ecology was found to be key to maintaining the desired outcomes in adolescents such as improved physical and mental health, cognitive development and educational attainment [19,20]. Therefore, the aim of MST intervention is to support caregivers to implement quality and effective parenting skills, because this has been shown to improve the overall well-being of adolescents that can consequently reduce the negative impact of socio-economic disadvantage [10,21].

The MST intervention model is based on nine treatment principles serving as guidelines for therapeutic assessments and treatment practices [22]. These are as follows: (*1*) *Clinicians should carry out assessments aimed at understanding how identified problems ‘fit’ within the broader context of the individual’s social ecology;* (*2*) *clinicians should focus on the positive, and utilise systemic strengths as levers for change;* (*3*) *interventions should be designed to promote responsible behaviour and decrease irresponsible behaviour among family members;* (*4*) *interventions should be present-focused and action-oriented, targeting specific and well-defined problems;* (*5*) *interventions should target sequences of behaviour within and between multiple systems that maintain identified problems;* (*6*) *interventions should be developmentally appropriate and fit the developmental needs of the young person;* (*7*) *interventions should be designed to require daily or weekly effort by family members;* (*8*) *intervention effectiveness should be evaluated continuously from multiple perspectives;* (*9*) *interventions should be designed to promote treatment generalisation and long-term maintenance of therapeutic change*.

To assess a clinician’s adherence to the nine principles of MST, the Therapist Adherence Measure (TAM-R) was implemented as a tool to measure clinician treatment fidelity within a quality assurance process. Treatment fidelity is defined as the extent to which the therapeutic intervention is delivered as it was designed. It refers to therapy accountability, including the quality of treatment delivery, the supervisory practices that support treatment implementation and the clinician’s ability to adhere to the practice protocol during therapy sessions [23]. Many studies that have been conducted by both MST model developers and independent researchers have demonstrated that when the treatment model was implemented correctly (i.e., with high levels of prescribed treatment fidelity), the effectiveness of the intervention was high [24,25,26,27,28].

The Western Australian Child and Adolescent Mental Health Service’s Multisystemic Therapy (MST) program is delivered by two small clinical teams located within the Perth city metropolitan area. Each clinical team is comprised of one clinical supervisor and four full-time clinicians when fully staffed. The therapy is intensive, with a clinician having a concurrent caseload of only four to six families whom they usually visit three times each week in the family home or community. The length of the service is typically 4–6 months with clinicians available 24/7 to support caregivers in times of family disruption and distress during the intervention. The majority of families referred to WA CAMHS MST are socio-economically disadvantaged and experiencing a wide range of complex and challenging issues. A significant proportion of referred families identify as ethnic minority, i.e., Aboriginal and Torres strait islander, or Culturally and Linguistically Diverse (CALD) families. These families often have a history of failed therapeutic interventions, and/or minimal positive contact with mental health and other social support services. The WA CAMHS MST service has consistently focused on building positive relationships with other departments and community services to effectively reach out to these marginalised populations. This results in successfully re-engaging youth in educational/vocational settings, reducing drug and alcohol use, lowering risk of homelessness and preventing further involvement with the Police and Justice Departments. Successful youth engagement intervention requires a robust working relationship with youth’s caregivers and other significant individuals [29]. Therefore, the MST approach facilitates this essential initial family engagement phase by closely working with families within their own homes and communities, at mutually suitable times, including after normal working hours.

The main goal of the MST intervention is to enhance the caregiver’s ability to support their adolescent with warm communication, whilst establishing firm and consistent boundaries, which in turn result in decreased adolescent disruptive behaviours. To achieve this goal, the clinicians work consistently within the MST therapeutic model, and the Therapist Adherence Measure (TAM-R) is implemented independently as a therapy compliance measurement tool. Previous published studies have confirmed that therapist adherence to the MST model is a valid predictor for a reduction of behavioural problems in adolescents; however, there is limited understanding of the mechanisms by which therapist model adherence is related to positive changes in family relations, and how these changes associate with decreased adolescent behavioural problems.

The aims of this retrospective study were firstly to examine how the therapist adherence to the MST treatment model affects family outcomes. Secondly, the study aimed to investigate the interaction between the change in parental outcomes (i.e., parental mental health, discipline approaches and monitoring skill), and improvement of adolescent emotional and behavioural problems after the MST intervention. Since prior research has shown that caregivers’ mental health issues may affect their ability to effectively implement learned parenting skills, we aimed to investigate the mediating effect of caregivers’ mental health on the relationship between improved parenting approaches and adolescent behavioural outcomes.

As a result, we formulated the following hypotheses: H1: Therapist adherence to the treatment model would predict positive change in parental mental health, discipline approaches and monitoring skills, as well as change in adolescent behavioural outcomes at post-treatment. H2: Positive changes in parental monitoring and discipline approaches would lead to improved parental mental health, which would then reduce adolescent behavioural problems post-treatment. We hope information gained from this study is used to inform programs and practitioners working with at-risk adolescents and families, to understand the mechanisms that may impact the effectiveness of the MST interventions.

## 2. Method

### 2.1. Participants and Procedure

This retrospective study extracted the data collected from 186 families who participated in this program’s research project during 2018–2024. The majority of referrals were made by schools and healthcare providers while a relatively smaller number were made by the department of community services (e.g., child protection and family services, and juvenile justice services). All families who had consented for the MST program operating within the Western Australian Child and Adolescent Mental Health Service (CAMHS) were offered to participate in the research project. They were assured their decision to participate in the research was voluntary, and that they could withdraw at any time. A written informed consent was obtained from caregivers once they agreed to participate in the research project. Then, a face-to-face interview was rescheduled with caregivers by research staff at two different time points (i.e., baseline and post-treatment). The therapist adherence measure scores were collected via a structured telephone interview made monthly during the intervention. Instruments used for the data collection contained questionnaires as well as face-to-face semi-structured interviews. This research project was approved by W.A. Health Central Human Research Ethics Committee.

### 2.2. Measures

#### 2.2.1. Child Behaviour Checklist (CBCL/6-18)

CBCL/6-18 is used to measure emotional and behavioural problems in adolescents, and their social competence. The parent-reported version was administered in this study to monitor changes in adolescents’ emotional and behavioural problems over time. There are eight subscales, comprising the following: anxious/depressed, withdrawn, somatic complaint, social problems, thought problems, attention problems, rule-breaking behaviour, aggressive behaviour and other problems. These subscales are categorised into two main components: internalising behaviours and externalising behaviours. The measure consists of 113 items scored on a 3-point Likert scale: not at all (0), somewhat true (1) and very true (2). The scale has high psychometric properties with internal reliability (Chronbach’s α) of 0.97 for total empirically based problem scales and the alphas of each subscales ranging from 0.79 to 0.97 [30]. Higher CBCL scores indicated higher levels of adolescent emotional and behavioural problems. The total raw scores were converted into standardised T-scores to assess the percentage of adolescents in the clinical ranges before and after the MST treatment. The T-scores above 63 are considered in the clinical range.

#### 2.2.2. Depression, Anxiety and Stress Scale-21 (DASS-21)

DASS-21 was also reported by caregivers. It contains three self-report subscales designed to measure the negative emotional states of depression, anxiety and stress [31]. DASS-21 used for this research is an abbreviated version with three subscales (depression, anxiety and stress) of 7 items each. The internal reliability (α-value) for the standardised 7-item scales is 0.81 for depression, 0.73 for anxiety and 0.81 for stress. The score for each subscale is determined by totalling the scores of the 7 corresponding items and multiplying it by 2. These scores were used to determine the severity of the caregiver’s depression, anxiety and stress. Higher DASS-21 scores indicated greater levels of caregiver’s mental health difficulties.

#### 2.2.3. Parenting Styles and Dimensions Questionnaire (PSDQ)

PSDQ was reported by caregivers and used to measure parenting styles in line with Baumrind’s Typology [32]. The measure categorised parenting styles into 3 dimensions: authoritative, authoritarian and permissive parenting styles [33]. The PSDQ contains 32 statements describing different caregiver’s responses to their adolescent behaviours. It has a 5-point scale ranging from ‘never’ to ‘always’ to evaluate the frequency of certain parenting strategies and reactions. The statements cover three dimensions of authoritative parenting (connection, regulation and autonomy) with an internal reliability (α-value) of 0.86, three dimensions of authoritarian parenting (physical coercion, verbal hostility and non-reasoning/punitive) with an internal reliability of 0.82 and one dimension of permissive parenting (indulgence) with an internal reliability of 0.64. Higher PSDQ scores indicated more frequent caregiver implementation of these parenting approaches.

#### 2.2.4. Parental Monitoring Scale (PM)

The Parental Monitoring scale used was adapted from an existing scale developed by Stattin and Kerr [34], which includes 23 questions using 5-point Likert scales ranging from never to always. There are 4 subscales composed of child disclosure (CD, 5 items), parental solicitation (PS, 5 items), parental control (PC, 5 items) and parental knowledge (PK, 8 items). This self-report scale asks caregivers about knowledge of their child’s whereabouts, activities and associations, as well as how information about their child was acquired (e.g., “How often do you know: what your child is doing during their free time? With whom your child is spending their free time? What your child spends their money on?”). The internal reliability (α-value) for each subscale was 0.84 for child disclosure, 0.75 for parental solicitation, 0.77 for parental control and 0.89 for parental knowledge. Higher scores indicated higher level of parental monitoring.

#### 2.2.5. Therapist Adherence Measure (TAM-R)

The Therapist Adherence Measure Revised developed by Henggeler et al. [35] is a standardised measure, composed of 28 items based on the nine treatment principles of MST. The measure evaluates a therapist’s adherence to the MST therapeutic model as reported by the primary caregiver. As part of MST operational quality assurance, TAM-R is collected monthly via a semi-structured telephone interview during the 4- to 5-month duration of the treatment. It has a five-point scale ranging from ‘not at all’ to ‘very much’. The rating of ‘very much’ is the only response rated ‘adherent’. Internal reliability (α-value) for components within the scale ranged from 0.86 to 0.91 [36]. The adherence score is calculated by dividing the number of items rated as adherent by number of items that can be scored. The adherence score ranges from 0 to 1, with 1 as the highest score of adherences. Scores lower than the threshold of 0.61 indicate poor adherence.

### 2.3. Data Analytic Strategy

Extracted data were analysed using the statistical software SPSS version 29. Socio-demographic data were summarized using descriptive statistics (e.g., mean, standard deviation) for continuous variables and frequencies (absolute and relative) for categorical variables. Paired-samples *t*-tests were used to investigate the change of adolescent and parental outcomes at baseline and post-treatment. Some families had withdrawn consent for research participation at post-treatment, so only cases with completed baseline and post-treatment data were included in the analysis.

**Figure 1:**
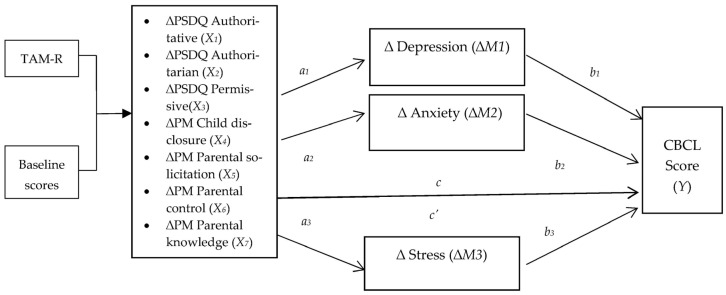
A conceptual diagram of the path analysis with the parallel multiple mediator model.

For the main analyses, we subtracted the post-treatment scores from the baseline score for CBCL, DASS, PSDQ authoritarian and PSDQ permissiveness; on the other hand, we subtracted the baseline scores from the post-treatment scores for parental monitoring (i.e., child disclosure, parental solicitation, parental control and parental knowledge) and PSDQ authoritative. The scores’ subtraction resulted in change scores (∆). Higher change scores indicated greater improvement in parental and adolescent outcomes. We tested the first hypothesis that the TAM-R score would directly predict changes on parental mental health, discipline approaches and monitoring skill as well as the changes on adolescent emotional and behavioural problems using linear regression analysis. The effects were controlled for scores at baseline. To test the second hypothesis that changes in DASS-21, parental monitoring (PM) and discipline approaches (PSDQ) have direct and indirect effects on adolescent emotional and behavioural problems, the parallel multiple mediator model was performed using PROCESS V3.4 developed by Andrew F. Hayes [37]. The parallel multiple mediator model aims to test the hypothesis that the influence of changes on discipline approaches and monitoring skill (*X_i_*) on adolescent emotional and behavioural problems (*Y*) would be mediated by changes on parental mental health (i.e., depression (*M*_1_), anxiety (*M*_2_) and stress (*M*_3_)). Figure 1 depicts a process in which the independent variables led to the mediators and the mediators then led to the dependent variable. With *k* = 3 mediators, four equations are needed:

(1) $$M_{1}=i_{M1}+a_{1}Xi+e_{M1}\;M_{2}=i_{M2}+a_{2}\mathrm{Xi}+e_{M2}\;M_{3}=i_{M3}+a_{3}\mathrm{Xi}+e_{M3}$$

(2) $$Y=i_{Y}+c\prime X+b_{1}M_{1}+b_{2}M_{2}+b_{3}M_{3}+e_{Y}$$

## 3. Results

### 3.1. Preliminary Analyses

Out of 186 families who agreed to research participation at baseline, 147 families (79%) remained at post-treatment data collection, and were included in the subsequent analysis. There was no statistically significant difference between study dropouts and study completers with respect to CBCL, DASS-21, parental monitoring (PM) and PSDQ scores at baseline. The independent sample *t*-test comparing study dropouts and completers resulted as follows: CBCLinternalising t(184) = −1.208, *p* > 0.05; CBCLexternalising t(184) = 0.474, *p* > 0.05; CBCLtotal t(184) = −0.265, *p* > 0.05; DASSstress t(184) = 1.331, *p* > 0.05; DASSanxiety t(184) = 0.298, *p* > 0.05; DASSdepression t(184) = 1.123, *p* > 0.05; PMchild disclousure t(181) = −0.240, *p* > 0.05; PMparent solicitation t(181) = −1.304, *p* > 0.05; PMparental control t(181) = 0.316, *p* > 0.05; PMparental knowledge t(181) = −4.28, *p* > 0.05; PSDQauthoritative t(181) = −1.309, *p* > 0.05; PSDQauthoritarian t(181) = 0.493, *p* > 0.05; PSDQpermissiveness t(181) = −0.696, *p* > 0.05.

The mean age of adolescents was 13.8 years (SD = 1.51, range 11–16 years), and 77% (n = 112) of adolescents were male. Majority of adolescents were identified as Caucasian (86%), 11% as Culturally and Linguistically Diverse (CALD) and 3% as Australian Aboriginal. Less than half of these adolescents (43%) lived with an intact family, 38% lived with a single caregiver, 12% with a blended family and 7% lived with caregivers who were not biological parents (e.g., foster parents, grandparents, or relatives). More than half of caregivers (58%) had their highest level of education in a high school or had a trade certificate, and 42% had a university degree. Around one third of families (34%) had an annual income, not including welfare benefits, of less than AUD 50,000 per annum. Around half of the adolescents had used illicit drugs or alcohol at least once in the previous 6 months (mostly alcohol and cannabis). We also discovered that many adolescents had recent adverse childhood experiences (ACEs) including parental separation and/or divorce (54.8%), family income significantly decreased (46.7%), parent lost job (31%), family deeply in debt (19%), alcohol or drug problem in family (47%), legal problem (44%), death of close relative or friend (39%), and having a family member with persistent physical illness (43%) or mental health issues (91%).

The results from converting CBCL raw scores to the standardised T-score show that at baseline, the percentage of adolescents falling within the clinical range was 86% for internalising behaviours, 97% for externalising behaviours and 97% for total behaviours; in comparison, post-treatment percentages were 57%, 71% and 72%, respectively. We also found that 93% (n = 137) of caregivers reported average TAM-R scores above the threshold of 0.61 which indicated that most families perceived their clinicians to have an adequate to high level of MST model adherence during the intervention period. The *t*-test results show there were statistically significant differences between baseline and post-treatment in all scores indicating improvements in parental and adolescent outcomes (Table 1).

### 3.2. Predictors of Changes in Parental and Adolescent Outcomes

The initial correlation analysis showed links between TAM-R and changes in adolescent outcomes (Supplementary Table S2). For parental outcomes, only the change in authoritarian score was found to have a significant correlation with TAM-R. The inter-correlations between changes in CBCL and changes in parental outcomes were found to be statistically significant in parental depression, anxiety, stress, parental monitoring (PM) child disclosure, PM parental control, PM parental knowledge, PSDQ authoritative, PSDQ authoritarian and PSDQ permissiveness. Linear regression models were then performed to test whether TAM-R predicted changes in parental and adolescent outcomes after adjusting for baseline scores. The results showed that a higher TAM-R score was associated with more improvements in CBCL, PM child disclosure, PSDQ authoritarian and PSDQ permissiveness after the treatment. Furthermore, we found that adolescents with more severe CBCL scores at baseline experienced a greater reduction in CBCL scores following the treatment. Caregivers who scored lower in child disclosure at baseline reported a stronger increase in child disclosure after the treatment, whereas caregivers who scored higher in authoritarian and permissiveness at baseline reported a greater reduction in these scores after the treatment. Table 2 shows only the six linear regression models with significant effects. Model 1 explained 18.3% of the variance (*R*^2^*_Adjusted_* = 0.172), Model 2 explained 15.7% of the variance (*R*^2^*_Adjusted_* = 0.145), Model 3 explained 22.6% of the variance (*R*^2^*_Adjusted_* = 0.215), Model 4 explained 14.5% of the variance (*R*^2^*_Adjusted_* = 0.133), Model 5 explained 39.4% of the variance (*R*^2^*_Adjusted_* = 0.386) and Model 6 explained 22.7% of the variance (*R*^2^*_Adjusted_* = 0.216).

### 3.3. Path Analysis and Mediating Effect Findings

**Figure 2:**
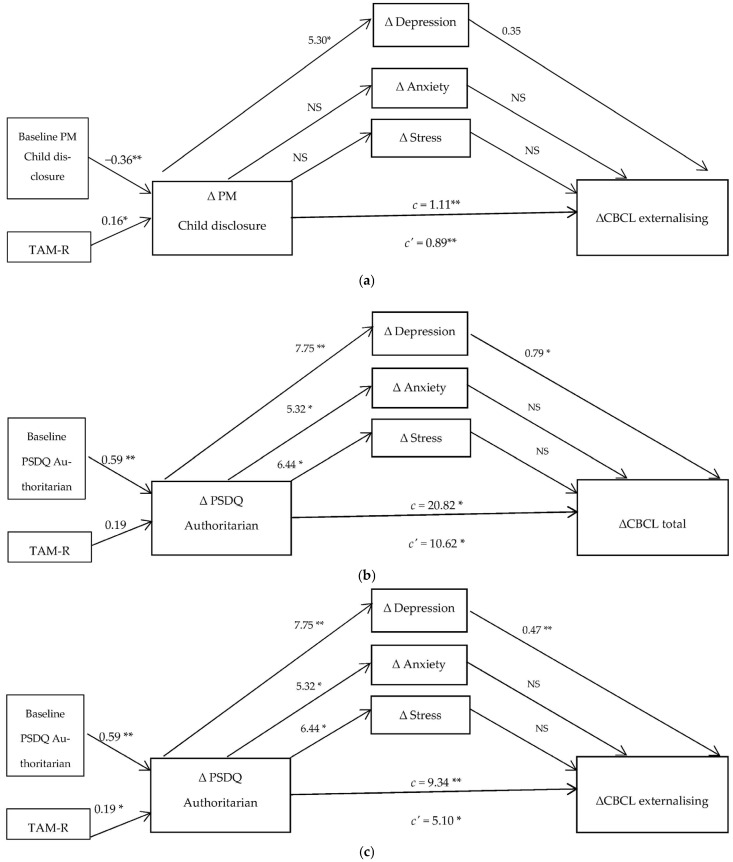

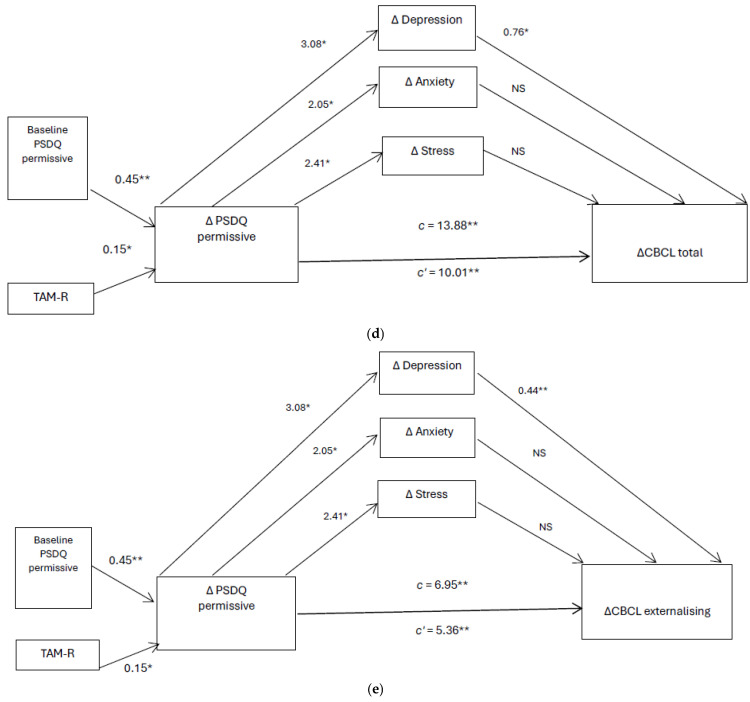
(**a**) Depicting Model a, an effect of TAM on changes in caregiver’s monitoring skill (i.e., child disclosure) predicted changes on adolescent externalising behavioural problems with change in parental depression as a mediator. Note: *c* = total effect, *c′* = direct effect, * *p* < 0.05, ** *p* < 0.01, NS = not statistically significant. (**b**) Depicting Model b, an effect of TAM on changes in caregiver’s authoritarian predicted changes on adolescent Total behavioural problems with change in parental depression as a mediator. Note: *c* = total effect, *c′* = direct effect, * *p* < 0.05, ** *p* < 0.01, NS = not statistically significant. (**c**) Depicting Model c, an effect of TAM on changes in caregiver’s authoritarian predicted changes on adolescent externalising behavioural problems with change in parental depression as a mediator. Note: *c* = total effect, *c′* = direct effect, * *p* < 0.05, ** *p* < 0.01, NS = not statistically significant. (**d**) Depicting Model d, an effect of TAM on changes in caregiver’s permissiveness predicted changes on adolescent Total behavioural problems with change in parental depression as a mediator. Note: *c* = total effect, *c′* = direct effect, * *p* < 0.05, ** *p* < 0.01, NS = not statistically significant. (**e**) Depicting Model e, an effect of TAM on changes in caregiver’s permissiveness predicted changes on adolescent externalising behavioural problems with change in parental depression as a mediator. Note: *c* = total effect, *c′* = direct effect, * *p* < 0.05, ** *p* < 0.01, NS = not statistically significant.

The parallel multiple mediator models (Table 3 and Figure 2a–e) illustrate the total (*c*), direct (*c′*) and indirect (*a_i_b_i_*) effects of changes in parental discipline approaches and monitoring skill on changes in adolescent behavioural problems, with changes in parental mental health as mediating variables. Only the models with statistical significance on the mediating effect are presented. Model a (Figure 2a) depicts the indirect effects of change in child disclosure on CBCL externalizing behaviours through mediating variables, estimated (*a_i_b_i_*) as follows: depression = 0.183, anxiety = 0.003 and stress = 0.031. Model b (Figure 2b) depicts the indirect effects of change in authoritarian approach on CBCL total problem behaviours through mediating variables, estimated (*a_i_b_i_*) as follows: depression = 6.146, anxiety = 1.022 and stress = 3.03. Model c (Figure 2c) depicts the indirect effects of change in authoritarian approach on CBCL externalising behaviours through mediating variables, estimated (*a_i_b_i_*) as follows: depression = 3.629, anxiety = −0.133 and stress = 0.738. Model (d) depicts the indirect effects of change in permissiveness on CBCL total problem behaviours through mediating variables, estimated (*a_i_b_i_*) as follows: depression = 2.30, anxiety = 0.380 and stress = 1.161. Model e (Figure 2e) depicts the indirect effects of change in permissiveness on CBCL externalising behaviours through mediating variables, estimated (*a_i_b_i_*) as follows: depression = 1.367, anxiety = −0.062 and stress = 0.286.

Table 3 shows around a third of the variance in changes in adolescent externalising and total problem behaviours were accounted for by proposed mediators (i.e., parental stress, anxiety and depression) and independent variables (i.e., discipline approaches and monitoring skill). The indirect effect pathways indicated there were significant associations found between changes in parental authoritarian, permissiveness and monitoring skill, and changes in parental mental health (i.e., anxiety, stress and depression). Change in parental depression was predicted by change in child disclosure (R^2^ = 0.05), change in authoritarian approach (R^2^ = 0.09) and change in permissiveness (R^2^ = 0.05). Change in parental anxiety was predicted by change in authoritarian approach (R^2^ = 0.06) and change in permissiveness (R^2^ = 0.03). Change in parental stress was predicted by change in authoritarian approach (R^2^ = 0.07) and change in permissiveness (R^2^ = 0.03). Positive change in adolescent externalising behaviours was found to be predicted by positive change in parental depression. Change in parental anxiety and stress was slightly associated with adolescent behavioural problems; however, it was not statistically significant.

## 4. Discussion

The results of the preliminary analyses indicated that many adolescents who were referred to MST were from families of low socio-economic status and had recent exposure of adverse childhood experiences (ACES), e.g., parental separation, significant loss of family income, alcohol or drug problems in the family, persistent physical or mental illness of family member. The results were aligned with prior empirical studies indicating a strong influence of ACES on increased risk of internalising and externalising behaviours in children and adolescents [38,39]. It is important to take into consideration the level of ACEs experienced by adolescents when working with families to address barriers impacting the desired family outcomes. The results also demonstrated that most caregivers positively perceived their MST clinician to adhere to the MST therapeutic model. Positive changes were reported in adolescents’ emotional and behavioural problems, and in caregivers’ mental health, parenting and monitoring skills after their involvement with the MST intervention.

In line with our first hypothesis, we found that therapist adherence to the MST treatment model positively predicted changes in adolescent internalising and externalising behaviours, and changes in parental monitoring (in child disclosure), authoritarianism and permissiveness at post-treatment. This finding is consistent with previous studies investigating the impact of therapist adherence on MST effectiveness on reducing adolescent problem behaviours and improving family functioning [24,25,27,40,41]. We also found that baseline scores in parenting and adolescent factors influenced the degree of changes of family outcomes. When TAM-R score increased, a stronger reduction in adolescent problem behaviours was found in adolescents who presented with high levels of behaviour problems at baseline. Similar results also appeared in parenting outcomes. Caregivers with high levels of authoritarianism, permissiveness and low levels of child disclosure monitoring approach at baseline were more likely to report stronger positive changes in their parenting approaches when the TAM-R score increased. In other words, with high levels of MST model adherence, caregivers with poor parenting skills at baseline are more likely to report greater benefit from the intervention compared to caregivers with moderate to high levels of parenting skills. This could be explained by previous findings by Dekovic et al. [42] showing that successful implementation of MST enhanced growth in parental sense of competence and positive discipline leading to improved adolescent outcomes. Therefore, caregivers with poor parenting skills at baseline would experience a greater shift in their ability after MST intervention compared to caregivers who already have adequate skills before MST. This outcome implies that MST intervention can be implemented successfully for families with severe problems.

It is notable that we did not find a direct effect of the therapist adherence on changes in parental depression, anxiety and stress. Despite finding improvement of parental mental health at post-treatment, the TAM-R score was not a direct indicator of these changes. This could be explained by the fact that the main goal of MST intervention is to improve parent–child interaction, empower caregivers to effectively communicate and establish clear and healthy boundaries with their adolescent [27] which was in line with MST principles, and can be measured by TAM-R. The caregiver’s mental health issues would be addressed only if it became a barrier in the clinician’s efforts to facilitate these caregiver skills. Clinicians work collaboratively with the family using systemic strengths as drivers for change. Therefore, an improvement in parental mental health was more likely to be a secondary gain from improved relationship between the parent–child and caregiver’s sense of efficacy and competence. The caregiver’s improved mental health was not directly linked to TAM-R score but indirectly through changes in their parenting approaches. To confirm this reasoning, the second hypothesis was tested to explore the mediating effects of parental mental health on the relationship between changes in a caregiver’s parenting approaches and improvement in adolescent problem behaviours.

The results from the mediation analyses confirmed both direct and indirect effects between changes in parental monitoring (i.e., child disclosure), authoritarianism, permissiveness and improvement in adolescent behavioural problems. The direct effects show that changes in the caregiver’s monitoring skill (i.e., child disclosure), authoritarianism and permissiveness directly predicted changes in adolescent outcomes. The indirect associations demonstrated that a change in parental depression partially mediated the relationship between changes in parental monitoring, discipline approaches and changes in adolescent problem behaviours. Positive changes in child disclosure, authoritarianism and permissiveness predicted an improvement in caregiver’s depression, and subsequently was associated with reductions in adolescent externalizing and total behavioural problems. It is worth noting that although there were three parental monitoring approaches (i.e., child disclosure, parental solicitation and parental control) tested in this study, TAM-R score only predicted an increase in child disclosure which consequently was associated with improved adolescent outcomes. This indicated that not all monitoring approaches were equally effective in adolescents with antisocial behaviours. Child disclosure, which happens when adolescents spontaneously and voluntary tell parents about their activities, peers and whereabouts, is more likely to be effective when high levels of trust and good communication between caregivers and adolescents are fostered [43]. This is congruent with previous studies showing that willingness of adolescents to disclose their activities was a critical factor to parenting monitoring [44,45]. The other monitoring approaches like parental solicitation (parents directly questioning their child, peers and peer’s parents about their child’s whereabout, activities and peers) and parental control (parents enforced rules and limit setting on child’s activities and peers) could work well with caregivers who have already established a warm and supportive relationship with their adolescent [46]. Conversely, these approaches might be perceived by adolescents with antisocial problems as invasive and controlling, resulting in further conflict between caregivers and adolescents especially when they are experiencing ongoing emotional disconnection [47]. Hayes, Hudson and Matthew (2003) [43] suggested that when the parent–adolescent relationship is poor, the initial step in improved monitoring would be rebuilding their relationship, rather than forcefully eliciting information about their adolescent’s activities and punishing them for not following rules.

We also found that caregivers who reported increased child disclosure and decreased authoritarianism and permissiveness were more likely to report decreased depression, anxiety and stress level at post-treatment. However, only a decrease in caregiver’s depression contributed to an improvement in adolescent externalising and total problem behaviours. The result is consistent with previous studies indicating that improvements in a caregiver’s mood was associated with increased treatment effectiveness in reducing adolescent emotional and behavioural problems [48,49]. These findings are congruent with other studies [10,11], indicating that caregivers experiencing poor mental health are more prone to employing negative parenting strategies (e.g., physical punishment, verbal hostility and/or avoidance) than those with healthier mental well-being. Our outcomes indicate that improved parenting skills had a positive impact on parental mental well-being which improve their child’s behaviours and general functioning. This supports the notion that achieving positive improvements in a caregiver’s parenting skills and mental health are strong indicators of desired treatment outcomes [48,50,51].

The results of this study should be viewed in consideration of some methodological limitations. Due to an absence of a control or comparison group, and only two time points being measured and analysed, we cannot rule out the possibility that there might be some confounding effect of natural variations over time. The results also indicated that only around a third of the variance in reduction of adolescent problem behaviours was accounted for by proposed mediators (parental mental health), and independent variables (i.e., child disclosure, authoritarianism and permissiveness). This suggested that there were other confounding factors contributing to improved adolescent behavioural problems during the intervention that should be further investigated. We recommend further research to examine mechanisms of change in MST therapy by investigating other plausible drivers such as family socio-cultural backgrounds, impact of adverse childhood experience, client’s willingness to change, clinician skills and expertise, therapeutic relationships and clinician/client compatibility. Identification of potential contributors to mechanisms of therapeutic change is a key to successful implementation of the MST intervention.

There are also limitations in the instruments used for this study. All measures were solely based on the caregiver’s ratings which are subject to informant bias. Inclusion of multi-informant measures, e.g., adolescent self-reported, teacher-reported and clinician-reported measures, would provide more diverse perspectives, identify discrepancies or validate the findings, resulting in a more comprehensive examination. Further evaluation using longitudinal data to track changes over time is strongly recommended to address potential confounding variables, and to help identify more complex causal relationships. Despite these limitations, this study provides substantial evidence indicating how MST model implementation fidelity, improved parental monitoring and discipline approaches resulted in improved caregiver’s mental well-being and consequently influenced positive outcomes in adolescent behaviour and functioning. This finding is congruent with recommendations from previous research [52,53] indicating that although randomised control trials (RCTs) are the gold standard for evaluating the effectiveness of the therapy program, they could be redundant for an intervention like MST where multiple RCTS have already been reported in the past 30 years. Those studies recommended that instead of repeatedly conducting RCTs to confirm MST treatment effectiveness, further research should focus on examining underlying mechanisms of MST effectiveness which are essential for planning policy, and providing evidence-based recommendations for clinical practice guidelines [1].

## 5. Conclusions

The MST intervention facilitates caregivers to develop effective communication skills and implement well-defined parenting strategies for managing antisocial behaviours while promoting pro-social behaviours in their adolescent. Therefore, the caregiver’s capacity to apply newly acquired skills needs to be accessed, and any barriers to its success needs to be addressed consistently during the intervention. The therapeutic relationship between the clinician and caregivers enhances an alignment in therapy and motivates caregivers to pursue continual supports for their own mental well-being. We believe the findings of this study are essential for refining interventions and optimising their effectiveness by identifying the most influential mechanisms involved. The outcomes from this study confirm previous findings that with the right combination of family and social supports, caregivers experiencing mental health challenges can improve their own well-being, develop effective parenting skills and strengthen their relationships with their children [1,9,11]. Also, these learned strategies are designed to be adaptable and flexible so that caregivers can overcome new challenging behaviours that might emerge in the future or support their other children. The MST intervention thus has the potential to generate a significant and lasting positive impact on society which could lead to substantial cost savings for the larger community.

## Figures and Tables

**Table 1:** Paired samples *t*-test results of CBCL, DASS, parental monitoring and PSDQ (N = 147).

Variable	Paired Differences								
	Pre-Treatment		Post-Treatment		95% CI of *Mdiff*		*t* (146)	*p*	Cohen’s *d*
	*µ*	(*SD*)	*µ*	(*SD*)	LL	UL			
**CBCL**									
Internalising problems	25.11	10.81	17.25	10.74	6.36	9.36	10.35	<0.001	0.85
Externalising problems	40.42	10.86	26.27	13.42	12.13	16.19	13.78	<0.001	1.14
Total problems	102.60	28.46	69.63	31.89	28.37	37.57	14.16	<0.001	1.17
**DASS**									
Depression	15.47	10.82	8.60	8.87	5.52	8.75	7.80	<0.001	0.72
Anxiety	11.89	9.83	6.63	8.08	3.78	6.73	7.05	<0.001	0.58
Stress	20.39	9.88	13.26	9.07	5.13	8.61	8.73	<0.001	0.64
**Parental Monitoring (PM)**									
Child Disclosure (CD)	10.87	4.56	13.44	5.18	−3.30	−1.83	−6.88	<0.001	−0.57
Parental Solicitation (PS)	16.39	3.31	17.33	3.85	−1.44	−0.33	−3.31	<0.05	−0.26
Parental Control (PC)	19.66	4.40	20.72	3.88	−1.64	−0.47	−6.93	<0.001	−0.29
Parental Knowledge (PK)	27.84	6.78	30.82	6.61	−3.79	−2.10	−7.24	<0.001	−0.57
**PSDQ**									
Authoritarian	1.93	0.48	1.56	0.40	0.30	0.43	10.96	<0.001	0.90
Permissiveness	3.02	0.81	2.30	0.81	0.60	0.84	11.70	<0.001	0.97
Authoritative	3.77	0.54	4.02	0.55	−0.32	−0.18	−7.24	<0.001	−0.61

**Table 2:** Linear regression models (N = 147).

Measure	B	β	t	95% CI		*p*
				LL	UL	
Model 1 DV = ∆CBCL total Baseline CBCL total TAMR	0.363 46.45	0.366 0.219 R^2^ = 0.183,	4.85 2.91 R^2^_adjusted_ = 0.172	0.215 14.88	0.510 78.02	<0.001 0.004
Model 2 DV = ∆CBCL external Baseline CBCL external TAMR	0.389 18.55	0.339 0.198 R^2^ = 0.157,	4.43 2.59 R^2^_adjusted_ = 0.145	0.216 4.40	0.562 32.70	<0.001 0.011
Model 3 DV = ∆CBCL internal Baseline CBCL internal TAMR	0.370 13.64	0.434 0.197 R^2^ = 0.226,	5.92 2.69 R^2^_adjusted_ = 0.215	0.246 3.62	0.493 23.65	<0.001 0.008
Model 4 DV = ∆PM Child Disclosure Baseline PM Child Disclosure TAMR	−0.358 5.28	−0.363 0.155 R^2^ = 0.145,	−4.70 2.01 R^2^_adjusted_ = 0.133	−0.509 0.099	−0.208 10.45	<0.001 0.046
Model 5 DV = ∆PSDQ authoritarian Baseline PSDQ authoritarian TAMR	0.503 0.591	0.590 0.191 R^2^ = 0.394,	9.09 2.94 R^2^_adjusted_ = 0.386	0.394 0.194	0.612 0.988	<0.001 0.004
Model 6 DV = ∆PSDQ permissive Baseline PSDQ permissive TAMR	0.420 0.840	0.454 0.148 R^2^ = 0.227,	6.20 2.03 R^2^_adjusted_ = 0.216	0.286 0.020	0.554 1.66	<0.001 0.045

DV = dependent variable, PM = parental monitoring. ∆ = change score.

**Table 3:** Regression coefficients, standard errors and model summary information for the parallel multiple mediator model (n = 147).

	Consequent																					
		*M*_1_ (∆Dep)				*M*_2_ (∆Anx)				*M*_3_ (∆Str)				*Y* (∆CBCL Total)			*Y* (∆CBCL Int)			*Y* (∆CBCL Ext)		
		Coeff.	*SE*	*P*		Coeff.	*SE*	*P*		Coeff.	*SE*	*P*		Coeff.	*SE*	*P*	Coeff.	*SE*	*P*	Coeff.	*SE*	*P*
*X*_1_ (∆PM CD)	*a*_1_	0.530	0.191	0.006	*a*_2_	0.130	0.166	0.435	*a*_3_	0.143	0.182	0.432	*c′*	1.796	0.435	<0.001	0.424	0.157	0.008	0.893	0.192	<0.001
*M*_1_ (∆Dep)		-	-	-		-	-	-		-	-	-	b_1_	0.551	0.294	0.063	0.084	0.106	0.429	0.345	0.130	0.008
*M*_1_ (∆Anx)		-	-	-		-	-	-		-	-	-	b_2_	0.293	0.300	0.329	0.131	0.108	0.227	0.023	0.132	0.861
*M*_1_ (∆Str)		-	-	-		-	-	-		-	-	-	b_3_	0.686	0.319	0.033	0.206	0.115	0.075	0.219	0.141	0.122
Constant	i_M1_	5.511	0.992	<0.001	i_M2_	4.925	0.860	<0.001	i_M3_	6.768	0.942	<0.001	i_Y_	18.137	2.527	<0.001	4.033	0.910	<0.001	7.808	1.117	<0.001
		R^2^ = 0.050 F(1,145) = 7.687, *p* < 0.05				R^2^ = 0.004 F(1,145) = 0.613, *p* = 0.435				R^2^ = 0.004 F(1,145) = 0.622, *p* = 0.432				R^2^ = 0.366 F(4,142) = 20.467, *p* < 0.001			R^2^ = 0.226 F(4,142) = 10.37 *p* < 0.001			R^2^ = 0.364 F(4,142) = 20.298, *p* < 0.001		
*X*_2_ (∆Authoritarian)	*a*_1_	7.754	2.079	<0.001	*a*_2_	5.32	1.79	0.004	*a*_3_	6.44	1.947	0.001	*c′*	10.62	5.08	0.039	2.427	1.791	0.177	5.104	2.280	0.027
*M*_1_ (∆Dep)		-	-	-		-	-	-		-	-	-	b_1_	0.793	0.298	0.009	0.141	0.105	0.180	0.468	0.133	<0.001
*M*_1_ (∆Anx)		-	-	-		-	-	-		-	-	-	b_2_	0.192	0.313	0.539	0.107	0.110	0.332	−0.025	140	0.858
*M*_1_ (∆Str)		-	-	-		-	-	-		-	-	-	b_3_	0.471	0.330	0.156	0.155	0.116	0.184	0.115	0.148	0.439
Constant	i_M1_	3.911	1.144	<0.001	i_M2_	3.218	0.985	0.001	i_M3_	4.666	1.071	<0.001	i_Y_	19.070	2.834	<0.001	4.277	0.998	<0.001	8.322	1.269	<0.001
		R^2^ = 0.088 F(1,144) = 13.904, *p* < 0.001				R^2^ = 0.058 F(1,144) = 8.808, *p* < 0.05				R^2^ = 0.071 F(1,144) = 10.931, *p* < 0.05				R^2^ = 0.304 F(4,141) = 15.429, *p* < 0.001			R^2^ = 0.191 F(4,141) = 8.323, *p* < 0.001			R^2^ = 0.288 F(4,141) = 14.225, *p* < 0.001		
*X*_3_ (∆Permissive)	*a*_1_	3.077	1.157	0.009	*a*_2_	2.049	0.990	0.040	*a*_3_	2.411	1.081	0.027	*c′*	10.008	2.609	<0.001	1.650	0.947	0.084	5.362	1.145	<0.001
*M*_1_ (∆Dep)		-	-	-		-	-	-		-	-	-	b_1_	0.757	0.287	0.009	0.141	0.104	0.179	0.444	0.126	<0.001
*M*_1_ (∆Anx)		-	-	-		-	-	-		-	-	-	b_2_	0.186	0.302	0.540	0.108	0.110	0.327	−0.030	0.132	0.820
*M*_1_ (∆Str)		-	-	-		-	-	-		-	-	-	b_3_	0.482	0.319	0.133	0.159	0.116	0.172	0.119	0.140	0.397
Constant	i_M1_	4.547	1.206	<0.001	i_M2_	3.698	1.031	<0.001	i_M3_	5.298	1.126	<0.001	i_Y_	15.934	2.852	<0.001	3.950	1.035	<0.001	6.475	1.251	<0.001
		R^2^ = 0.047 F(1,144) = 7.070, *p* < 0.05				R^2^ = 0.029 F(1,144) = 4.284, *p* < 0.05				R^2^ = 0.033 F(1,144) = 4.974, *p* < 0.05				R^2^ = 0.351 F(4,141) = 19.036, *p* < 0.001			R^2^ = 0.198 F(4,141) = 8.689, *p* < 0.00			R^2^ = 0.362 F(4,141) = 19.955, *p* < 0.001		

∆ = change score (baseline – post), PM = parental monitoring, CD = child disclosure, Dep = depression, Anx = anxiety, Str = stress.

## Data Availability

The data that support the findings of this study are the property of the Department of Health, Western Australia, therefore are not publicly available due to their containing information that could compromise the privacy of research participants. As a result, non-identifiable data are only available from the corresponding author upon reasonable request.

## Supplementary Materials

The following supporting information can be downloaded at https://www.mdpi.com/article/10.3390/ijerph22081310/s1, Figure S1a: depicting Model a, an effect of TAM on changes in caregiver’s monitoring skill (i.e., child disclosure) predicted changes on adolescent externalising behavioral problems with change in parental depression as a mediator; Figure S1b: depicting Model b, an effect of TAM on changes in caregiver’s authoritarian predicted changes on adolescent Total behavioral problems with change in parental depression as a mediator; Figure S1c: depicting Model c, An effect of TAM on changes in caregiver’s authoritarian predicted changes on adolescent externalising behavioral problems with change in parental depression as a mediator; Figure S1d: depicting Model d, an effect of TAM on changes in caregiver’s permissiveness predicted changes on adolescent Total behavioral problems with change in parental depression as a mediator; Figure S1e: depicting Model e, an effect of TAM on changes in caregiver’s permissiveness predicted changes on adolescent externalising behavioral problems with change in parental depression as a mediator; Table S1: Paired samples T-test results of CBCL, DASS, Parental monitoring and PSDQ; Table S2: Mean, standard deviations and correlations; Table S3: Linear regression models; Table S4: Regression coefficients, standard errors, and model summary information for the parallel multiple mediator model.
