# Supplementary material for: The Influence of Therapist Adherence on Multisystemic Therapy Treatment Outcome for Adolescents with Antisocial Behaviours: A Retrospective Study in Western Australian Families
Source: Int J Environ Res Public Health. 2025 Aug 21;22(8):1310. doi: 10.3390/ijerph22081310 (PMC12386259; doi:10.3390/ijerph22081310)

Supplementary tables and figures

Table S1. Paired samples T-test results of CBCL, DASS, Parental monitoring and PSDQ (*N* = 147).

Variable	Paired Differences								
	**Pre-treatment**		**Post-treatment**		**95% CI of *Mdiff***		***t(146)***	***p***	**Cohen's *d***
	μ	*(SD)*	*μ*	*(SD)*	Lower	Upper			
**CBCL**									
Internalising problems	25.11	10.81	17.25	10.74	6.36	9.36	10.35	<.001	.85
Externalising problems	40.42	10.86	26.27	13.42	12.13	16.19	13.78	<.001	1.14
Total problems	102.60	28.46	69.63	31.89	28.37	37.57	14.16	<.001	1.17
**DASS**									
Depression	15.47	10.82	8.60	8.87	5.52	8.75	7.80	<.001	.72
Anxiety	11.89	9.83	6.63	8.08	3.78	6.73	7.05	<.001	.58
Stress	20.39	9.88	13.26	9.07	5.13	8.61	8.73	<.001	.64
**Parental Monitoring (PM)**									
Child Disclosure (CD)	10.87	4.56	13.44	5.18	-3.30	-1.83	-6.88	<.001	-.57
Parental Solicitation (PS)	16.39	3.31	17.33	3.85	-1.44	-.33	-3.31	<.05	-.26
Parental Control (PC)	19.66	4.40	20.72	3.88	-1.64	-.47	-6.93	<.001	-.29
Parental Knowledge (PK)	27.84	6.78	30.82	6.61	-3.79	-2.10	-7.24	<.001	-.57
**PSDQ**									
Authoritarian	1.93	.48	1.56	.40	.30	.43	10.96	<.001	.90
Permissiveness	3.02	.81	2.30	.81	.60	.84	11.70	<.001	.97
Authoritative	3.77	.54	4.02	.55	-.32	-.18	-7.24	<.001	-.61

Table S2. Mean, standard deviations and correlations (N=147).

Variables	*M*	*SD*	1	2	3	4	5	6	7	8	9	10	11	12	13
**1. TAM-R**	.80	.13													
**2. ΔCBCLInt**	7.86	9.20	.19*												
**3. ΔCBCLExt**	14.26	12.45	.21*	.60**											
**4. ΔCBCLTot**	32.97	28.22	.22**	.86**	.89**										
**5. ΔStress**	7.14	9.92	.13	.40**	.43**	.48**									
**6. ΔAnxiety**	5.26	9.05	.10	.36**	.35**	.42**	.69**								
**7. ΔDepression**	6.87	10.68	.10	.40**	.51**	.52**	.76**	.66**							
**8. ΔPM Child Disclosure**	2.56	4.52	.12	.25**	.40**	.36**	.07	.07	.22**						
**9. ΔPM Parental Solicitation**	.93	3.40	.08	.41	.11	.09	.04	-.05	.14	.40**					
**10. ΔPM Parental Control**	1.06	3.62	.04	.07	.32**	.24**	.20*	.13	.32**	.36**	.36**				
**11. ΔPM Parental Knowledge**	2.98	5.22	.05	.26**	.41**	.35**	.22**	.11	.29**	.38**	.45**	.51**			
**12. ΔPSDQ Permissiveness**	.73	.75	.15	.22**	.42**	.37**	.18*	.17*	.22**	.19*	.201*	.33**	.24**		
**13. ΔPSDQ Authoritarian**	.37	.41	.22**	.23**	.31**	.30**	.27**	.24**	.30**	.40	.07	.23**	.12	.47**	
**14. ΔPSDQ Authoritative**	.25	.41	.10	.25**	.25**	.25**	.18*	.08	.25**	.32**	.19*	.29**	.22**	.10	.15

Δ = Change score, PM = parental monitoring, PSDQ = parenting style

*** = p < .05, ** = p <.001**

Table S3. Linear regression models (N=147).

Measure	B	β	t	95%CI		*P*
				LL	UL	
Model 1						
DV=ΔCBCL total						
Baseline CBCL total	.363	.366	4.85	.215	.510	<.001
TAMR	46.45	.219	2.91	14.88	78.02	.004
		R^2 = .183,	$R^2_{adjusted}$ = .172			
Model 2						
DV=ΔCBCL external						
Baseline CBCL external	.389	.339	4.43	.216	.562	<.001
TAMR	18.55	.198	2.59	4.40	32.70	.011
		R^2 = .157,	$R^2_{adjusted}$ = .145			
Model 3						
DV=ΔCBCL internal						
Baseline CBCL internal	.370	.434	5.92	.246	.493	<.001
TAMR	13.64	.197	2.69	3.62	23.65	.008
		R^2 = .226,	$R^2_{adjusted}$ = .215			
Model 4						
DV=ΔPM Child Disclosure						
Baseline PM Child Disclosure	-.358	-.363	-4.70	-.509	-.208	<.001
TAMR	5.28	.155	2.01	.099	10.45	.046
		R^2 = .145,	$R^2_{adjusted}$ = .133			
Model 5						
DV=ΔPSDQ authoritarian						
Baseline PSDQ authoritarian	.503	.590	9.09	.394	.612	<.001
TAMR	.591	.191	2.94	.194	.988	.004
		R^2 = .394,	$R^2_{adjusted}$ = .386			
Model 6						
DV=ΔPSDQ permissive						
Baseline PSDQ permissive	.420	.454	6.20	.286	.554	<.001
TAMR	.840	.148	2.03	.020	1.66	.045
		R^2 = .227,	$R^2_{adjusted}$ = .216			

DV = dependent variable, PM = parental monitoring. Δ = change score.

Table S4. Regression coefficients, standard errors, and model summary information for the parallel multiple mediator model (n = 147).

		**Consequent**																				
		***M_1* (ΔDep)**				***M_2* (ΔAnx)**				***M_3* (ΔStr)**				***Y (ΔCBCL total)***			***Y (ΔCBCL int)***			***Y (ΔCBCL ext)***		
		**Coeff.**	***SE***	***P***		**Coeff.**	***SE***	***P***		**Coeff.**	***SE***	***P***		**Coeff.**	***SE***	***P***	**Coeff**	***SE***	***P***	**Coeff.**	***SE***	***P***
X_1 (ΔPM CD)	a_1	.530	.191	.006	a_2	.130	.166	.435	a_3	.143	.182	.432	c'	1.796	.435	<.001	.424	.157	.008	.893	.192	<.001
M_1 (ΔDep)		-	-	-		-	-	-		-	-	-	b_1	.551	.294	.063	.084	.106	.429	.345	.130	.008
M_1 (ΔAnx)		-	-	-		-	-	-		-	-	-	b_2	.293	.300	.329	.131	.108	.227	.023	.132	.861
M_1 (ΔStr)		-	-	-		-	-	-		-	-	-	b_3	.686	.319	.033	.206	.115	.075	.219	.141	.122
Constant	i_{M1}	5.511	.992	<.001	i_{M2}	4.925	.860	<.001	i_{M3}	6.768	.942	<.001	i_Y	18.137	2.527	<.001	4.033	.910	<.001	7.808	1.117	<.001
		$R^2 = .050$				$R^2 = .004$				$R^2 = .004$				$R^2 = .366$			$R^2 = .226$			$R^2 = .364$		
		$F(1,145)=7.687$,				$F(1,145)= .613$,				$F(1,145)= .622$,				$F(4,142)=20.467$,			$F(4,142)=10.37$			$F(4,142)=20.298$,		
		$p < .05$				$p = .435$				$p = .432$				$p < .001$			$p < .001$			$p < .001$		
X_2 (ΔAuthoritarian)	a_1	7.754	2.079	<.001	a_2	5.32	1.79	.004	a_3	6.44	1.947	.001	c'	10.62	5.08	.039	2.427	1.791	.177	5.104	2.280	.027
M_1 (ΔDep)		-	-	-		-	-	-		-	-	-	b_1	.793	.298	.009	.141	.105	.180	.468	.133	<.001
M_1 (ΔAnx)		-	-	-		-	-	-		-	-	-	b_2	.192	.313	.539	.107	.110	.332	-.025	.140	.858
M_1 (ΔStr)		-	-	-		-	-	-		-	-	-	b_3	.471	.330	.156	.155	.116	.184	.115	.148	.439
Constant	i_{M1}	3.911	1.144	<.001	i_{M2}	3.218	.985	.001	i_{M3}	4.666	1.071	<.001	i_Y	19.070	2.834	<.001	4.277	.998	<.001	8.322	1.269	<.001
		$R^2 = .088$				$R^2 = .058$				$R^2 = .071$				$R^2 = .304$			$R^2 = .191$			$R^2 = .288$		
		$F(1,144)=13.904$,				$F(1,144)=8.808$,				$F(1,144)=10.931$,				$F(4,141)=15.429$,			$F(4,141)=8.323$,			$F(4,141)=14.225$,		
		$p < .001$				$p < .05$				$p <.05$				$p < .001$			$p < .001$			$p < .001$		
X_3 (ΔPermissive)	a_1	3.077	1.157	.009	a_2	2.049	.990	.040	a_3	2.411	1.081	.027	c'	10.008	2.609	<.001	1.650	.947	.084	5.362	1.145	<.001
M_1 (ΔDep)		-	-	-		-	-	-		-	-	-	b_1	.757	.287	.009	.141	.104	.179	.444	.126	<.001
M_1 (ΔAnx)		-	-	-		-	-	-		-	-	-	b_2	.186	.302	.540	.108	.110	.327	-.030	.132	.820
M_1 (ΔStr)		-	-	-		-	-	-		-	-	-	b_3	.482	.319	.133	.159	.116	.172	.119	.140	.397
Constant	i_{M1}	4.547	1.206	<.001	i_{M2}	3.698	1.031	<.001	i_{M3}	5.298	1.126	<.001	i_Y	15.934	2.852	<.001	3.950	1.035	<.001	6.475	1.251	<.001
		$R^2 = .047$				$R^2 = .029$				$R^2 = .033$				$R^2 = .351$			$R^2 = .198$			$R^2 = .362$		
		$F(1,144)=7.070$,				$F(1,144)=4.284$,				$F(1,144)=4.974$,				$F(4,141)=19.036$,			$F(4,141)=8.689$,			$F(4,141)=19.955$,		
		$p < .05$				$p < .05$				$p <.05$				$p < .001$			$p < .00$			$p < .001$		

Δ = change score, PM = parental monitoring, CD = child disclosure, Dep = depression, Anx = anxiety, Str = Stress.

Figure S1a. depicting **Model a**, an effect of TAM on changes in caregiver's monitoring skill (i.e. child disclosure) predicted changes on adolescent externalising behavioral problems with change in parental depression as a mediator. Note: *c* = total effect, *c'* = direct effect, **p* <.05, ***p*<.01, NS = not statistically significant.

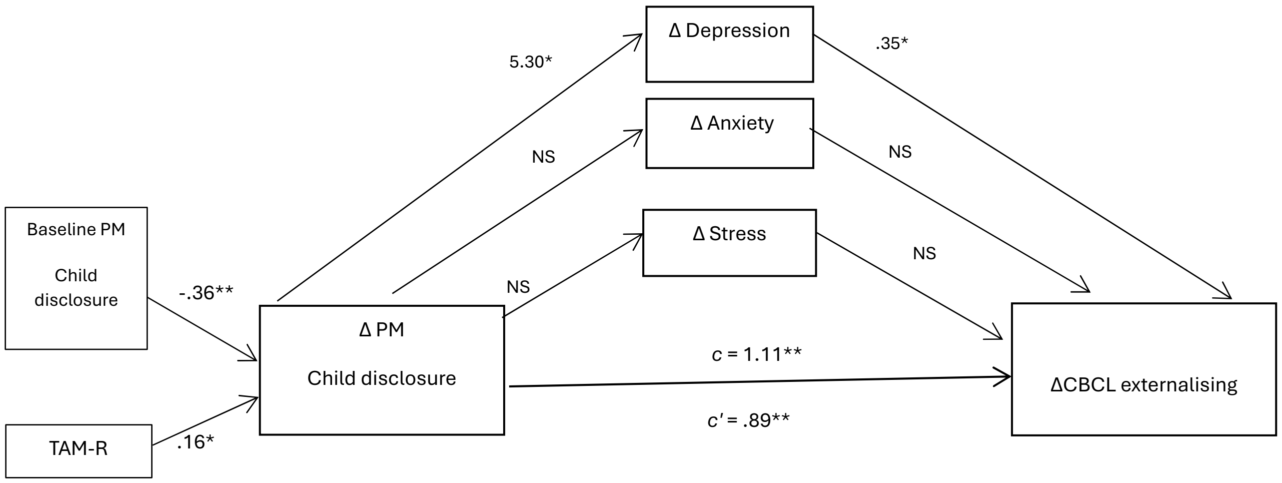

Figure S1b. depicting **Model b**, an effect of TAM on changes in caregiver's authoritarian predicted changes on adolescent Total behavioral problems with change in parental depression as a mediator. Note: *c* = total effect, *c'* = direct effect, **p* <.05, ***p*<.01, NS = not statistically significant.

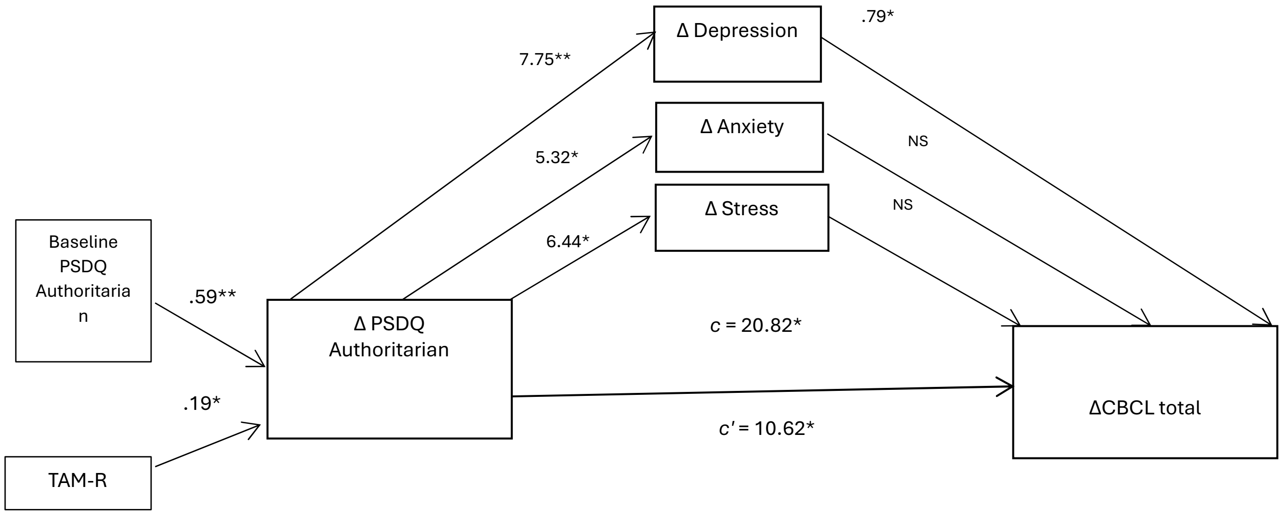

Figure S1c. depicting **Model c**, An effect of TAM on changes in caregiver's authoritarian predicted changes on adolescent externalising behavioral problems with change in parental depression as a mediator. Note: *c* = total effect, *c'* = direct effect, **p* <.05, ***p*<.01, NS = not statistically significant.

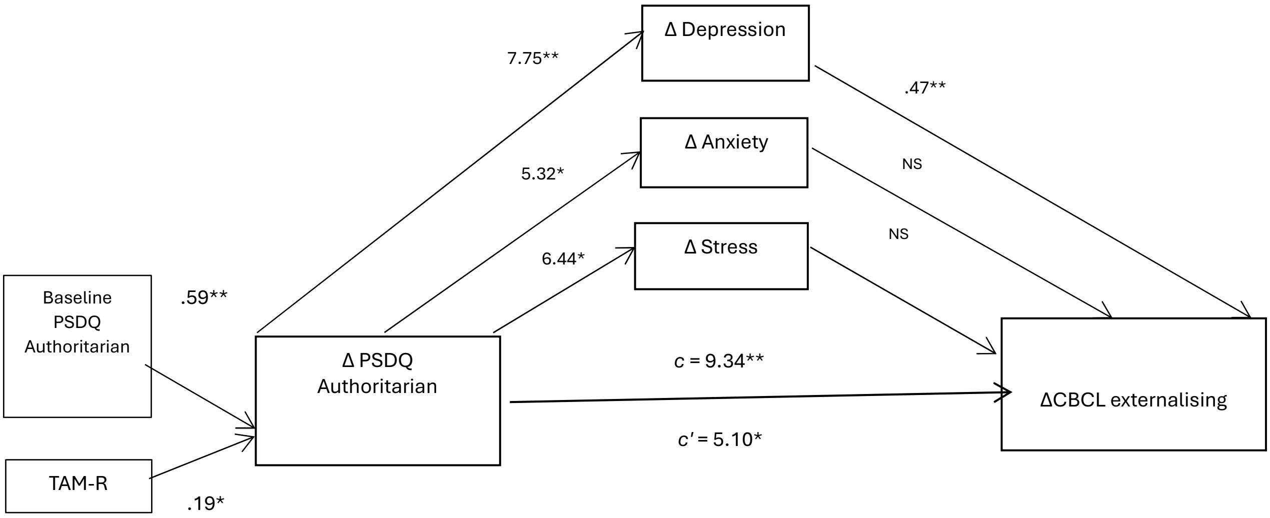

Figure S1d. depicting **Model d,** an effect of TAM on changes in caregiver's permissiveness predicted changes on adolescent Total behavioral problems with change in parental depression as a mediator. Note: *c* = total effect, *c'* = direct effect, **p* <.05, ***p*<.01, NS = not statistically significant.

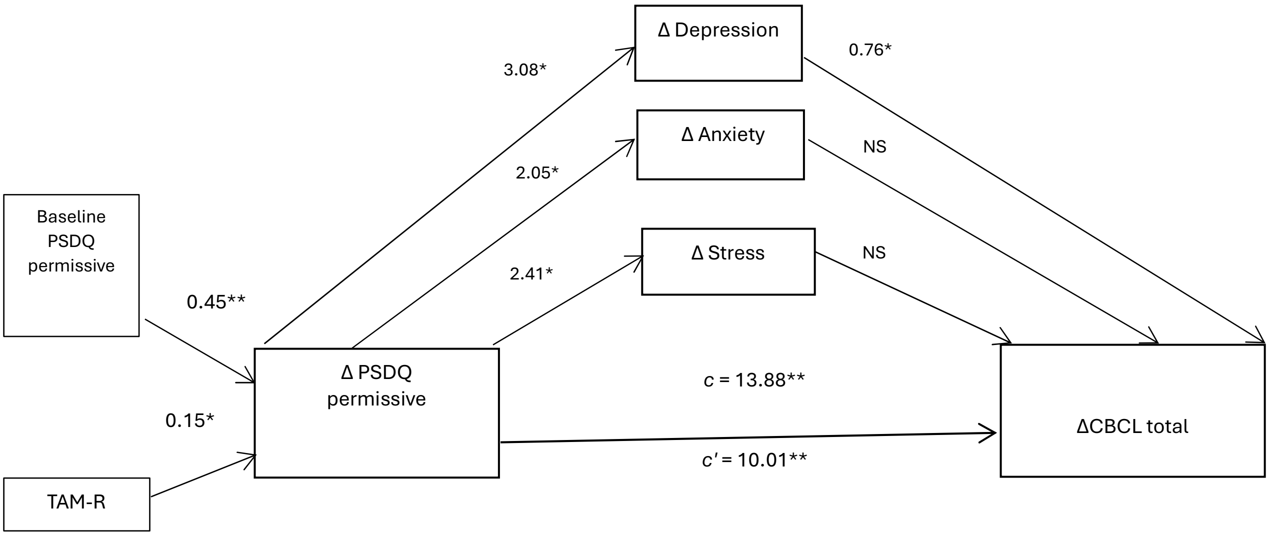

Figure S1e. depicting **Model e**, an effect of TAM on changes in caregiver's permissiveness predicted changes on adolescent externalising behavioral problems with change in parental depression as a mediator. Note: *c* = total effect, *c'* = direct effect, **p* <.05, ***p*<.01, NS = not statistically significant.

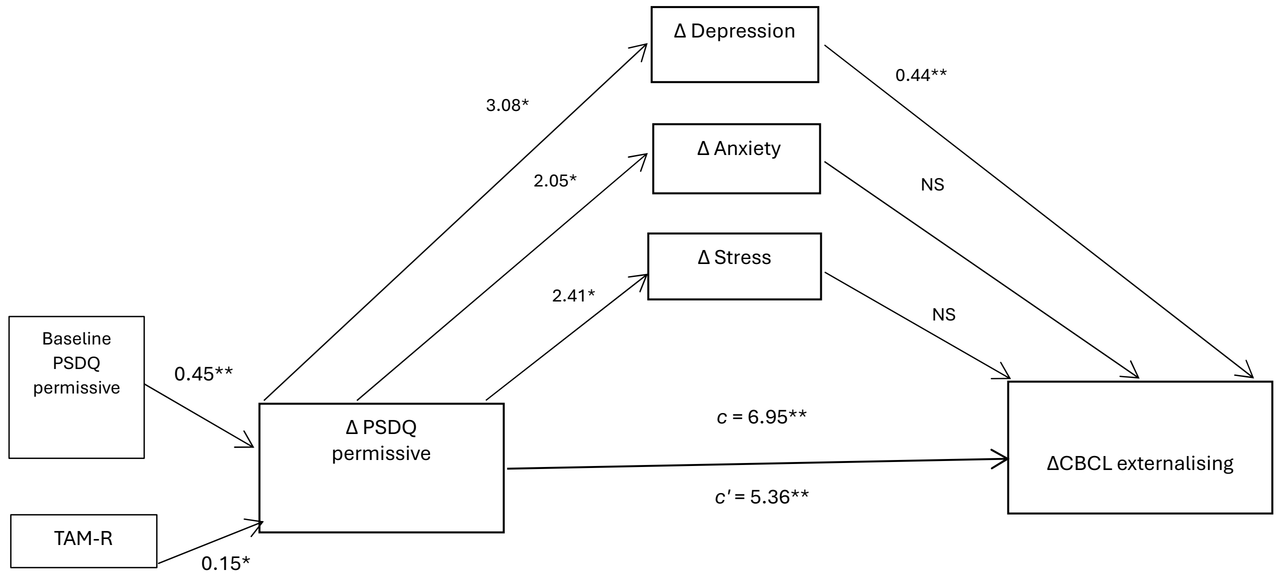

Supplement: Supplementary file 1 [file ijerph-22-01310-s001.zip › ijerph-3732133-supplementary.pdf]
